# Associations Between Vascular Risk Across Adulthood and Brain Pathology in Late Life: Evidence From a British Birth Cohort

**DOI:** 10.1001/jamaneurol.2019.3774

**Published:** 2019-11-04

**Authors:** Christopher A. Lane, Josephine Barnes, Jennifer M. Nicholas, Carole H. Sudre, David M. Cash, Ian B. Malone, Thomas D. Parker, Ashvini Keshavan, Sarah M. Buchanan, Sarah E. Keuss, Sarah-Naomi James, Kirsty Lu, Heidi Murray-Smith, Andrew Wong, Elizabeth Gordon, William Coath, Marc Modat, David Thomas, Marcus Richards, Nick C. Fox, Jonathan M. Schott

**Affiliations:** 1 Dementia Research Centre, UCL Queen Square Institute of Neurology, University College London, London, United Kingdom; 2 London School of Hygiene and Tropical Medicine, Department of Medical Statistics, University of London, London, United Kingdom; 3 School of Biomedical Engineering and Imaging Sciences, King’s College London, London, United Kingdom; 4 MRC Unit for Lifelong Health and Ageing at UCL, University College London, London, United Kingdom; 5 Leonard Wolfson Experimental Neurology Centre, UCL Queen Square Institute of Neurology, University College London, London, United Kingdom; 6 Neuroradiological Academic Unit, Department of Brain Repair and Rehabilitation, UCL Queen Square Institute of Neurology, University College London, London, United Kingdom; 7 UK Dementia Research Institute at UCL, University College London, London, United Kingdom

## Abstract

**Importance:**

Midlife vascular risk burden is associated with late-life dementia. Less is known about if and how risk exposure in early adulthood influences late-life brain health.

**Objective:**

To determine the associations between vascular risk in early adulthood, midlife, and late life with late-life brain structure and pathology using measures of white matter–hyperintensity volume, β-amyloid load, and whole-brain and hippocampal volumes.

**Design, Setting, and Participants:**

This prospective longitudinal cohort study, Insight 46, is part of the Medical Research Council National Survey of Health and Development, which commenced in 1946. Participants had vascular risk factors evaluated at ages 36 years (early adulthood), 53 years (midlife), and 69 years (early late life). Participants were assessed with multimodal magnetic resonance imaging and florbetapir-amyloid positron emission tomography scans between May 2015 and January 2018 at University College London. Participants with at least 1 available imaging measure, vascular risk measurements at 1 or more points, and no dementia were included in analyses.

**Exposures:**

Office-based Framingham Heart study–cardiovascular risk scores (FHS-CVS) were derived at ages 36, 53, and 69 years using systolic blood pressure, antihypertensive medication usage, smoking, diabetic status, and body mass index. Analysis models adjusted for age at imaging, sex, *APOE* genotype, socioeconomic position, and, where appropriate, total intracranial volume.

**Main Outcomes and Measures:**

White matter–hyperintensity volume was generated from T1/fluid-attenuated inversion recovery scans using an automated technique and whole-brain volume and hippocampal volume were generated from automated in-house pipelines; β-amyloid status was determined using a gray matter/eroded subcortical white matter standardized uptake value ratio threshold of 0.61.

**Results:**

A total of 502 participants were assessed as part of Insight 46, and 463 participants (236 male [51.0%]) with at least 1 available imaging measure (mean [SD] age at imaging, 70.7 [0.7] years; 83 β-amyloid positive [18.2%]) who fulfilled eligibility criteria were included. Among them, FHS-CVS increased with age (36 years: median [interquartile range], 2.7% [1.5%-3.6%]; 53 years: 10.9% [6.7%-15.6%]; 69 years: 24.3% [14.9%-34.9%]). At all points, these scores were associated with smaller whole-brain volumes (36 years: β coefficient per 1% increase, −3.6 [95% CI, −7.0 to −0.3]; 53 years: −0.8 [95% CI, −1.5 to −0.08]; 69 years: −0.6 [95% CI, −1.1 to −0.2]) and higher white matter–hyperintensity volume (exponentiated coefficient: 36 years, 1.09 [95% CI, 1.01-1.18]; 53 years, 1.02 [95% CI, 1.00-1.04]; 69 years, 1.01 [95% CI, 1.00-1.02]), with largest effect sizes at age 36 years. At no point were FHS-CVS results associated with β-amyloid status.

**Conclusions and Relevance:**

Higher vascular risk is associated with smaller whole-brain volume and greater white matter–hyperintensity volume at age 69 to 71 years, with the strongest association seen with early adulthood vascular risk. There was no evidence that higher vascular risk influences amyloid deposition, at least up to age 71 years. Reducing vascular risk with appropriate interventions should be considered from early adulthood to maximize late-life brain health.

## Key Points

**Question:**

When is vascular risk during adulthood (early adulthood, midlife, or late life) most strongly associated with late-life brain structure and pathology?

**Findings:**

In a propective cohort of 463 participants free of dementia from the population-based Insight 46 study, higher vascular risk in early adulthood was most strongly associated with smaller whole-brain volumes and greater white matter–hyperintensity volumes at age 69 to 71 years. There were no associations at any age with amyloid status.

**Meaning:**

These findings are consistent with vascular risk influencing late-life brain health via cerebral small-vessel disease and lower brain volumes but not amyloidosis; vascular risk screening and modification may need to be considered from early adulthood.

## Teaser

This longitudinal cohort study assesses the associations between vascular risk in early adulthood, midlife, and late life with late-life brain structure and β-amyloid load and white matter hyperintensity, whole-brain, and hippocampal volumes.

## Introduction

Dementia affects 44 million people worldwide, a number expected to triple by 2050.^1^ Vascular risk factors, including hypertension,^2^ obesity,^3^ diabetes,^4^ and smoking,^5^ are implicated in the development of late-life cognitive impairment. Midlife (considered to span ages 40 years to 65 years) rather than late-life risk exposure is generally considered more critical.^6^ However, there has been little investigation into the influence of vascular risk prior to midlife. Appropriate timing of vascular screening programs and interventions will be necessary to maximize benefits to cognitive health at both an individual and population level.

Vascular risk burden confers increased risk for clinically diagnosed vascular dementia and also Alzheimer disease (AD) dementia (albeit to a lesser extent).^7^ The pathological mechanisms by which vascular risk factors mediate cognitive decline are not well understood, with conflicting evidence over whether vascular risk burden directly enhances β-amyloid deposition,^8,9^ a cardinal feature of AD, in addition to its well-established role in cerebral small-vessel disease. The *APOE* ε4 allele, the most important genetic risk factor for development of sporadic AD, is thought to influence β-amyloid deposition via alterations in its clearance.^10^ Whether *APOE* ε4 also modulates the association of vascular risk factors with cerebral pathology, as has been previously suggested,^11^ remains to be clarified.

Individuals from the Medical Research Council National Survey of Health and Development (NSHD; the British 1946 birth cohort) have had vascular phenotyping since their 30s, and now a subset have had multimodal magnetic resonance imaging and β-amyloid imaging. This allowed us to investigate the influence of vascular risk exposure timing on brain structure and pathology at age 69 to 71 years, with a specific focus on cerebral small-vessel disease, β-amyloid deposition, and brain volumes, using measures from early adulthood, midlife, and early late life. We hypothesized that (1) the strongest association between vascular risk burden and late-life brain structure and pathology would be observed with midlife risk exposure and (2) the influence of vascular risk burden on brain structure and pathology would be modified by *APOE* ε4 allele status.

## Methods

### Study Design and Participants

Study participants were from Insight 46, a substudy of the NSHD that initially included 5362 individuals born throughout mainland Britain in a single week in 1946.^12^ Eligibility criteria for Insight 46^13^ (eMethods in the Supplement for more detail) and comparisons with the larger NSHD^14^ have previously been reported. A total of 502 participants attended data-collection sessions at University College London between May 2015 and January 2018, where they underwent detailed clinical, cognitive, and brain-imaging assessments.^13^

Ethical approvals for the wider NSHD study have been described.^15^ This study was approved by the Queen Square research ethics committee; all participants provided written informed consent.

### Procedures

Imaging was performed on a single Biograph mMR 3T positron emission tomography (PET)/magnetic resonance imaging (MRI) scanner (Siemens Healthcare), with simultaneous acquisition of dynamic PET/MRI data, including volumetric (1.1-mm isotropic) T1 and fluid-attenuated inversion recovery (FLAIR) sequences, at age 69 to 71 years.^13^ The β-amyloid burden was assessed using ^18^F-florbetapir (Amyvid). Positron emission tomography data was processed using an automated in-house processing pipeline including pseudo–computed tomography attenuation correction.^13^ The global standardized uptake value ratio was calculated from cortical regions of interest (including the lateral and medial frontal, anterior, and posterior cingulate; lateral parietal; and lateral temporal regions), normalized to eroded subcortical white matter. Positive or negative β-amyloid status was determined using a Gaussian mixture model applied to standardized uptake value ratio values, taking the 99th percentile of the lower (β-amyloid negative) Gaussian plot as the cut point (0.61).

The T1-weighted and FLAIR images underwent visual quality control, before processing using the following automated pipelines^13^: whole-brain volume (WBV) segmentation using Multi-Atlas Propagation and Segmentation,^16^ hippocampal volume (HV) using Similarity and Truth Estimation for Propagated Segmentations^17^ (both with manual editing if required), and total intracranial volume using SPM12 (Wellcome Centre for Human Neuroimaging).^18^ A validated, unsupervised automated algorithm, Bayesian model selection (BaMoS)^19^ was used to segment white matter hyperintensities (WMH) from T1/FLAIR images, followed by visual quality control and editing if required, generating a global WMH volume (WMHV) including subcortical gray matter but excluding infratentorial regions.

### Vascular Risk Factors and Other Co-Variates

Vascular risk factors have been measured at in-person visits since age 36 years (except serum cholesterol levels, which were only collected at ages 53 years and 69 years). Therefore, for the purposes of this analysis, office-based Framingham Heart Study–cardiovascular risk scores (FHS-CVS), which do not require serum cholesterol levels for calculation, were derived from measurements collected on home visits by research nurses when participants were age 36 years (early adulthood), 53 years (midlife), and 69 years (early late life), prior to their Insight 46 visit. The FHS-CVS provides a 10-year risk of cardiovascular events. It is made up of a weighted sum of age, sex, systolic blood pressure, antihypertensive medication usage (yes/no), history of diabetes (yes/no), current smoking (yes/no), and body mass index (BMI; calculated as weight in kilograms divided by height in meters squared).^20^ Seated blood pressure was measured in the upper arm twice after 5 minutes of rest. At age 36 years, a Random Zero sphygmomanometer (Hawksley) was used; at ages 53 years and 69 years, an HEM-705 automated digital oscillometric sphygmomanometer (Omron) was used. To ensure compatibility, published conversion equations were applied.^21^ The second systolic blood pressure measure was used for analyses, unless data were missing. Prescription medication usage was self-reported: at each point, individuals were categorized according to antihypertensive medication usage.^22^ Smoking status was defined by questionnaire: for participants aged 69 years, this was collected from a postal questionnaire they had completed at age 68 years, and if these data were missing, we collected this from questionnaires completed at age 60 to 64 years. Diabetes mellitus status was based on self-reported diagnosis at age 36 years, and at ages 53 and 69 years, it was based on self-reported diagnosis or a hemoglobin A_1C_ level of 6.5% or more (to convert to a proportion of total hemoglobin, multiply by 0.01).

For descriptive purposes, hypertension was defined as either a blood pressure of 140 over 90 mm Hg or a self-reported clinical diagnosis at each point. Obesity was defined as a BMI of 30 or more. Hypercholesterolemia status was defined as random serum cholesterol level of 193.4 mg/dL (5 mmol/L or more; to convert to mmol/L, multiply by 0.0259) and/or, at age 69 years, self-reported use of a cholesterol-lowering medication.

We performed *APOE* genotyping using standard techniques from samples available at age 53 years,^23^ or if missing, at age 69 to 71 years, and individuals were categorized as having or not having at least 1 *APOE* ε4 allele. Adult socioeconomic position was defined as nonmanual labor or manual labor, based on occupation at age 53 years, according to the United Kingdom Registrar General’s Classification of Occupations.

### Statistical Analysis

Analyses were performed in Stata version 14.1 (StataCorp). To be included, participants needed to be free of dementia per expert consensus informed by clinical history, informant history, and Mini-Mental State Examination (score ≥26)^24^ and have acceptable-quality amyloid positron emission tomography and magnetic resonance imaging, known *APOE* ε4 status, and complete vascular risk factors information at 1 or more points. For WMHV and brain-volume analyses, individuals with cortical infarcts inappropriately segmented (n = 5) or white matter pathologies not considered vascular in origin (eg, demyelination; n = 3), were excluded. For brain-volume analysis, individuals also required a useable amyloid scan.

Separate analyses were performed to investigate associations between FHS-CVS at each age and each imaging measure. Because of the nonnormal distribution of WMHV, generalized linear models using the gamma distribution and log link were used to investigate associations with WMHV. Logistic regression models were used to investigate associations with β-amyloid status, and linear regression was used to investigate associations with WBV and mean HV. Models were adjusted for sex, scanning age, adult socioeconomic position, *APOE* ε4 status and (for WMHV and brain-volume models) total intracranial volume. Differential influences of FHS-CVS on imaging outcomes by *APOE* ε4 status and sex were tested by introduction of appropriate interaction terms into models. We did not correct for multiple comparisons because we were interested in examining the association at each time point separately. For each imaging outcome, effect-size differences between points were investigated by including FHS-CVS at the 3 ages in a joint model and testing for an age interaction, accounting for clustering using robust standard errors.

Generalized linear models and linear regression model assumptions were confirmed by examination of residuals plotted against fitted values. Logistic regression model assumptions were also assessed with the Hosmer-Lemeshow test for goodness of fit. Model checking indicated no material violation of assumptions or highly influential data points. Statistical significance was set at *P* < .05.

In a sensitivity analysis, multiple imputation was used for the 66 individuals excluded because of missing covariate data (eMethods in the Supplement). A further exploratory analysis sought to investigate whether a higher number of vascular risk factors, rather than a weighted risk score, was associated with imaging measures. Because of the previously reported association between more midlife vascular risk factors (using current smoking, hypertension, diabetes, obesity, and elevated total cholesterol) and late-life β-amyloid deposition,^8^ we sought to replicate this finding and extend it to other imaging measures by categorizing the number of risk factors as 0, 1, or 2 or more. This analysis was only performed on participants at age 53 years and 69 years, since serum cholesterol was unavailable for participants at age 36 years.

## Results

Of 502 individuals assessed, 471 (93.4%) completed the imaging protocol; of these, 468 (93.2%) were free of dementia. A total of 463 participants (236 male participants [51.0%]; mean [SD] age at imaging, 70.7 [0.7] years) were included in the sample.

Following imaging processing and quality control, 455 individuals (90.6%) were available for amyloid analysis, 443 individuals (88.2%) for brain-volume analysis, and 451 (89.8%) for WMHV analysis; all of these participants had necessary covariate data. (eFigure 1 in the Supplement summarizes recruitment and available data.) Age at imaging was similar across individuals. Participant characteristics are summarized in Table 1, including numbers with available vascular profiles at each age. (eTables 1-3 in the Supplement show comparisons of characteristics between those with and without missing data.) Participants in Insight 46 had marginally lower FHS-CVS results than in the full NSHD cohort (median [interquartile range (IQR)]: age 36 years, 2.7% [1.5%-3.6%] vs 2.9% [1.7%-4.3%]; age 53 years, 10.9% [6.7%-15.6%] vs 12.2% [7.6%-18.8%]; age 69 years, 24.3% [14.9%-34.9%] vs 25.0% [15.3%-36.5%]; eTable 4 in the Supplement).

**Table 1.** Participant Characteristics Including Vascular Risk Profiles at Ages 36 Years, 53 Years, and 69 Years and Imaging Outcome Measures

Characteristic	No. (%)			
	Age at Home Visit, y			Insight 46 Imaging Assessment
	36	53	69	
Total, No.	418	449	450	463
Age, mean (SD), y	36.3 (0.2)	53.4 (0.2)	69.5 (0.2)	70.7 (0.7)
Male	213 (51.0)	229 (51.0)	232 (51.6)	236 (51.0)
Adult socioeconomic position				
Nonmanual labor	360 (86.1)	383 (85.3)	386 (85.8)	393 (84.9)
Manual labor	58 (13.9)	66 (14.7)	64 (14.2)	70 (15.1)
*APOE* ε4 carrier status^a^	128 (30.6)	129 (28.7)	130 (28.9)	137 (29.6)
Systolic blood pressure, mean (SD), mm Hg	120.3 (13.8)	133.6 (19.2)	132.4 (16.0)	NA
Use of antihypertensive medication	7 (1.8)	53 (11.8)	180 (40.0)	NA
Hypertension	66 (15.8)	207 (46.1)	253 (56.4)	NA
BMI, mean (SD)	23.7 (3.1)	26.9 (4.0)	27.6 (4.4)	NA
Obesity	13 (3.1)	77 (17.2)	117 (26.0)	NA
Current smoker	82 (19.6)	41 (9.1)	16 (3.6)	NA
Diabetes	1 (0.2)	13 (2.9)	47 (10.4)	NA
Hypercholesterolemia	NR	348 (86.4)	364 (80.0)	NA
Office-based FHS-CVS, median (IQR), %^b^	2.7 (1.5-3.6)	10.9 (6.7-15.6)	24.3 (14.9-34.9)	NA
No. of participants with vascular risk factors recorded^c^	NR	403	455	NA
No. of vascular risk factors^c^				
0	NR	27 (6.7)	42 (9.2)	NA
1	NR	165 (40.9)	141 (31.0)	NA
2	NR	147 (36.5)	174 (38.2)	NA
3	NR	60 (14.9)	82 (18.0)	NA
4	NR	4 (1.0)	16 (3.5)	NA
5	NR	0	0	NA
Amyloid-positive. No./total No. of participants (%)	NR	NR	NR	83/455 (18.2)
Global white matter hyperintensity volume, median (IQR), mL	NR	NR	NR	3.1 (1.6-6.8)
Total No. of participants	NR	NR	NR	451
Whole-brain volume, mean (SD), mL	NR	NR	NR	1100 (98)
Total No. of participants	NR	NR	NR	443
Mean hippocampal volume, mean (SD), mL	NR	NR	NR	3.1 (0.3)
Total No. of participants	NR	NR	NR	443
Total intracranial volume, mean (SD), mL	NR	NR	NR	1434 (132)
Total No. of participants	NR	NR	NR	451

Abbreviations: BMI, body mass index (calculated as weight in kilograms divided by height in meters squared); FHS-CVS, Framingham Heart Study–cardiovascular risk score; IQR, interquartile range; NA, not applicable; NR, not recorded.

^a^ Presence of 1 or 2 alleles.

^b^ The office-based FHS-CVS provides a 10-year risk of cardiovascular events (as a percentage). It is made up of a weighted sum of age, sex, systolic blood pressure, use of antihypertensive medication (yes/no), history of diabetes (yes/no), current smoking (yes/no), and BMI.

^c^ For the purposes of exploratory analyses, vascular risk factors included current smoker status, presence of obesity, presence of diabetes, raised total cholesterol, and presence of hypertension. Information on cholesterol was not available at age 36 years.

### Associations Between Vascular Risk Scores and Global WMHV at Age 69 to 71 Years

Higher vascular risk scores were associated with higher WMHV. The effect size increased the earlier the FHS-CVS was measured (exponentiated coefficients: age 36 years, 1.09 [95% CI, 1.01-1.18]; *P* = .04; age 53 years, 1.02 [95% CI, 1.00-1.04]; *P* = .03; age 69 years, 1.01 [95% CI, 1.00-1.02; *P* = .02; Table 2; Figure; eFigure 2 in the Supplement). There was a significant difference in the effect size by age of risk measurement (interaction *P* < .001). There was no evidence of a sex or *APOE* ε4 interaction.

**Table 2.** Associations Between Framingham Heart Study–Cardiovascular Risk Scores at Ages 36, 53, and 69 Years and White Matter Hyperintensity Volume, Amyloid Status, Whole-Brain Volume, and Mean Hippocampal Volume at Age 69 to 71 Years^a^

Age, y	White Matter–Hyperintensity Volume, mL			Amyloid Status			Whole-Brain Volume, mL			Mean Hippocampal Volume, ml		
	Participants, No.	Exponentiated Coefficient (95% CI)	*P* Value	Participants, No.	Adjusted Odds Ratio (95% CI)	*P* Value	Participants, No.	β Coefficient (95% CI)	*P* Value	Participants, No.	β Coefficient (95% CI)	*P* Value
36	407	1.09 (1.01-1.18)	.04	410	0.98 (0.79-1.21)	.85	399	–3.6 (–7.0 to –0.3)	.03	399	–0.03 (–0.05 to –0.004)	.02
53	438	1.02 (1.00-1.04)	.03	441	0.97 (0.92-1.02)	.19	430	–0.8 (–1.5 to –0.08)	.03	430	–0.0001 (–0.005 to 0.004)	.96
69	438	1.01 (1.00-1.02)	.02	442	0.99 (0.97-1.02)	.50	430	–0.6 (–1.1 to –0.2)	.003	430	0.0001 (–0.003 to 0.003)	.96

^a^ Coefficients represent the change in imaging outcomes measures per 1% increase in Framingham Heart Study–cardiovascular risk scores. For instance, a 1% increase in score at age 36 years is associated with 9% higher white matter–hyperintensity volume, 3.6-mL smaller whole-brain volume, and a 0.03-mL smaller mean hippocampal volume. All models are adjusted for sex, age at time of scanning, *APOE* ε4 status, adult socioeconomic position, and (where appropriate) total intracranial volume.

**Figure.**
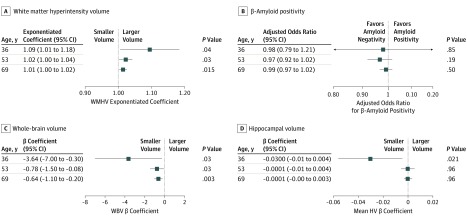
Plots Showing the Effect Sizes of a 1% Increase in Framingham Heart Study–Cardiovascular Risk Score at Ages 36, 53, and 69 Years on Imaging Outcome Measures at Age 69 to 71 Years HV indicates hippocampal volume; WBV, whole-brain volume; WMHV, white matter–hyperintensity volume.

### Associations Between Vascular Risk Scores and β-Amyloid Status at Age 69 to 71 Years

There was no association between vascular risk scores at any time point (ages 36, 53, or 69 years) and β-amyloid status at age 69 to 71 years (Table 2; Figure). Effect sizes did not differ between ages of vascular risk measurements. There was no evidence of sex or *APOE* ε4 interactions.

### Associations Between Vascular Risk Scores and Brain Volumes at Age 69 to 71 Years

Higher vascular risk scores were associated with smaller WBV, and the effect size increased the earlier the FHS-CVS was measured (β coefficient: age 36 years, –3.6 [95% CI, –7.0 to –0.3]; *P* = .03; age 53 years, –0.8 [95% CI, –1.5 to –0.08]; *P* = .03; age 69 years, –0.6 [95% CI, –1.1 to –0.2]; *P* = .003; Table 2; Figure). A higher FHS-CVS at age 36 years was associated with a smaller mean HV (β coefficient: age 36 years, –0.03 [95% CI, –0.05 to –0.004]; *P* = .02; Table 2; Figure). When WBV was included in this model, the association was no longer significant (β coefficient, −0.018 [95% CI, −0.038 to 0.003]; *P* = .09). Associations with WBV remained statistically significant when β-amyloid status and WMHV were introduced into models (β coefficient: age 36 years, –3.7 [95% CI, –7.1 to –0.3]; *P* = .03; age 53 years, –0.8 [95% CI, –1.5 to –0.07]; *P* = .03; age 69 years, –0.6 [95% CI, –1.1 to –0.2]; *P* = .003; eTable 5 in the Supplement). There was a significant difference in the effect size of vascular risk burden by age of risk measurement on WBV (β coefficients from interaction model: age 36 years, –3.7; age 53 years, –1.0; and age 69 years, –0.5; interaction *P* = .02) but not mean HV.

We initially observed a significant interaction between sex and FHS-CVS on WBV assessment at age 36 years only whereby higher vascular risk scores were associated with smaller WBV, but this only reached significance in women (β coefficient, −11.2 [95% CI, –19.3 to –3.0]; *P* = .008 in women; β coefficient, −2.1 [95% CI, −5.8 to 1.6]; *P* = .27 in men; interaction *P* = .048). There were no significant sex interactions observed at other ages or when examining mean HV. There were no significant interactions with *APOE*-ε4 status except at age 69 years, at which point an interaction between *APOE* ε4 status and FHS-CVS was initially observed on the expected mean HV, whereby in individuals carrying at least 1 *APOE* ε4 allele, an increasing vascular risk score at age 69 years was associated with a larger mean HV, while in those without this allele, an increasing score was associated with a smaller mean HV. However, in neither group was the association significant (those with at least 1 allele: β coefficient, 0.0028 [95% CI, −0.001 to 0.0067]; *P* = .15; those without at least 1 allele: β coefficient, −0.0015 [95% CI, −0.0046 to 0.0016]; *P* = .35; interaction *P* = .045).

### Associations Between Covariates and Brain Measures

Associations are summarized in eTable 6 in the Supplement. The individuals with at least 1 *APOE* ε4 allele were more likely to have β-amyloid positivity (odds ratio, 5.16 [95% CI, 3.02-8.81]; *P* < .001), but this was not associated with other imaging outcomes.

### Associations Between Imaging Measures and a Cumulative Vascular Risk Score

Increasing numbers of vascular risk factors at age 53 or 69 years were not associated with WMHV or brain volumes. The presence of 2 or more vascular risk factors at age 69 years was associated with a decreased likelihood of β-amyloid positivity (odds ratio, 0.40 [95% CI 0.18-0.91]; *P* = .03; Table 3).

**Table 3.** Associations Between Increasing Numbers of Vascular Risk Factors at Ages 53 and 69 Years and Cerebral Outcome Measures at Age 69 to 71 Years^a^

Factor	White Matter Hyperintensity Volume, mL		Amyloid Status		Whole-Brain Volume, mL		Mean Hippocampal Volume, mL	
	Exponentiated Coefficient (95% CI)	*P* Value	Adjusted Odds Ratio (95% CI)	*P* Value	β Coefficient (95% CI)	*P* Value	β Coefficient (95% CI)	*P* Value
Participants with vascular risk factor status at age 53 y, No.	393	NA	395	NA	385	NA	385	NA
Vascular risk factors								
None	[Reference]	NA	[Reference]	NA	[Reference]	NA	[Reference]	NA
1	1.12 (0.71-1.74)	.63	1.21 (0.37-3.91)	.76	–8.9 (–27.3 to 9.5)	.34	–0.02 (–0.14 to 0.11)	.78
≥2	1.40 (0.91-2.17)	.13	0.85 (0.26-2.75)	.79	–14.2 (–32.3 to 4.0)	.13	–0.04 (–0.17 to 0.08)	.47
Participants with vascular risk factor status at age 69 y, No.	443	NA	447	NA	435	NA	435	NA
Vascular risk factors								
None	[Reference]	NA	[Reference]	NA	[Reference]	NA	[Reference]	NA
1	0.94 (0.64-1.39)	.75	0.46 (0.19-1.11)	.08	6.2 (–9.9 to 22.2)	.45	0.09 (–0.02 to 0.19)	.10
≥2	1.20 (0.83-1.74)	.33	0.40 (0.18-0.91)	.03	–6.4 (–21.4 to 8.6)	.40	0.08 (–0.01 to 0.18)	.09

Abbreviation: NA, not applicable.

^a^ Models are adjusted for sex, adult socioeconomic position, APOE ε4 status, age at scanning, and (where appropriate) total intracranial volume.

### Sensitivity Analysis

Imputing missing FHS-CVS data (for 463 participants) did not significantly alter findings. Details are presented in eTable 7 in the Supplement.

## Discussion

In this study of individuals who were free of dementia, all of whom were nearly identical in age at the times of assessments, we demonstrate that the association of vascular risk with WMHV and brain volumes at age 69 to 71 years increases the earlier vascular risk is present, with the strongest influence at age 36 years. There was no evidence of an association between vascular risk and β-amyloid status when using the FHS-CVS score. These findings support the concept that vascular risk is associated with subsequent cognitive health through vascular pathways and influences on brain volume but not β-amyloid deposition at age 69 to 71 years. Furthermore, findings support that while midlife is an important period of risk exposure, influences of vascular risk on brain structure extend back into early adulthood and may be particularly damaging at this time or may alternatively reflect increased accumulated risk exposure.

Associations between vascular risk factors, cerebral small-vessel disease, smaller brain volumes, and cognitive impairment are widely reported.^25^ Although it is generally considered that midlife, rather than late-life risk exposure is more critical, direct comparisons between points are rarely reported. A recent study by the Framingham cohort used an associated composite vascular risk score (the Framingham Stroke Risk Profile) and demonstrated stronger associations between vascular risk burden at younger ages (as young as 45 years) and late-life brain volumes.^26^ We extend these findings by demonstrating that this temporal association exists with both WBV and WMHV, a proxy marker of cerebral small-vessel disease. Furthermore, the strongest associations were observed using measures at age 36 years, which is younger than previously reported. This is consistent with the recent observation of cross-sectional associations between cerebrovascular imaging markers and cardiovascular risk factors in young adults.^27^ Importantly, even at the low risk levels seen at this age, a 1% increase in risk can have a substantial influence 3 decades later. It should be noted that a 1% absolute increase in risk at age 36 years is a much larger relative increase in vascular risk than at later ages because of relatively low risk scores at that point. From a practical perspective however, even though a 10-year cardiovascular risk less than 10% is considered to be low risk, this study supports that changes in this range are still damaging to subsequent cerebral health.

We noted a stronger association between vascular risk and late-life brain volume in women at age 36 years only. A similar association in younger women only was reported by Pase et al,^26^ who suggested it may reflect individuals who are particularly unhealthy for their age, since high vascular risk is unusual in women who are premenopausal. However, in this cohort, the range of FHS-CVS risk scores in women were lower than those of men, with a similar spread around normal age-adjusted risk.^20^ A heightened vulnerability in women who are premenopausal might be associated with sex differences in vascular remodeling causing microvascular disease, which has been demonstrated in coronary artery disease.^28^ We did not observe a similar interaction when examining associations with WMHV. Since microvascular sequelae also include microinfarcts that influence atrophy,^29^ this association might be observed independently of conventional cerebral small-vessel disease imaging markers. Indeed, associations between vascular risk and WBV persisted with WMHV adjustment. Alternatively, the larger effect size in women might be associated with the underestimation of true cardiovascular risk in women when scores such as the FHS-CVS are used.^30^

We did not find a positive association between vascular risk scores and brain amyloidosis in late life. Previous findings are inconsistent: 1 study^31^ found a cross-sectional positive association between the Framingham coronary risk score and brain β-amyloid in older individuals, while a recent Atherosclerosis Risk in Communities study found an association with increasing number of vascular risk factors in midlife but not late life.^8^ Using a similar measure, we did not find an association with midlife vascular risk factors, which might be accounted for by cohort differences, including the younger age at imaging in Insight 46 (in the Atherosclerosis Risk in Communities study, the mean age was 76 years). Using a cumulative count of vascular risk factors in midlife and late life to investigate associations with brain volumes and WMHV did not replicate the findings using the FHS-CVS score, demonstrating the importance of using a validated, appropriately weighted score when assessing overall vascular risk.

In late life, however, we observed that a higher number of vascular risk factors was associated with decreased likelihood of β-amyloid-positivity. This may reflect a selection bias, since those who have a higher vascular burden and are also β-amyloid-positive may be more likely to be cognitively impaired and refuse participation. Alternatively, since BMI^32^ and blood pressure^33^ decline in the dementia prodrome, individuals with β-amyloid positivity may have lower vascular scores (ie, reverse causality).

In addition to the role of apolipoprotein E ε4 in enhancing β-amyloid deposition,^34^ it is involved in lipid metabolism and enhances atheroma deposition.^35^ It has been suggested that the negative association of vascular risk factors with brain pathology, including β-amyloid deposition,^36^ brain volumes,^37^ and WMHV,^38^ may be exacerbated in individuals who carry the *APOE* ε4 allele. However, we found no evidence of this. As anticipated, *APOE* ε4 carriage was strongly associated with β-amyloid–positive status, but there was no independent association with other imaging measures. This provides further evidence that the *APOE* ε4 allele influences late-life dementia risk through its association with β-amyloid deposition, while vascular risk influences late-life brain health through nonamyloidogenic pathways. Since many other pathological pathways are implicated in the development of AD, including neuroinflammation^39^ and tau-mediated damage,^40^ vascular risk may still directly influence AD risk via other mechanisms, which we were not able to investigate. However, an association with hippocampal volume was only seen at age 36 years, and this was substantially attenuated after WBV adjustment, suggesting this association was driven by global brain changes rather than a region-specific change, which might have been anticipated if vascular risk was directly associated with AD pathology.

We used a composite vascular score rather than examining vascular risk factors individually: vascular risk factors often cluster together, for instance, in the context of the metabolic syndrome (ie, obesity, hypertension, and diabetes),^41^ and therefore consideration of global cardiovascular risk is more relevant in clinical practice. We recently reported associations between higher blood pressure in midlife and greater WMHV and smaller brain volumes at age 69 to 71 years. Increasing blood pressure between age 36 years and 43 years (ie, early adulthood into midlife) was also associated with smaller brain volumes.^42^ In this analysis, incorporating information on other vascular risk factors may have increased the power to detect associations extending back into early adulthood, when vascular risk is generally low. Findings from this work have potential implications for public health strategies, highlighting the importance of vascular risk modification from early adulthood onwards to maximize benefits to late-life cognitive health. This is particularly pertinent in view of the rising global obesity epidemic, with associated consequent vascular effects.

### Limitations

Although participants are broadly representative of the population born in mainland Britain in 1946, those in Insight 46 are exclusively white British individuals, which limits generalizability to other populations. We have previously demonstrated that individuals in Insight 46 are healthier than in the larger NSHD.^14^ It is therefore likely that, if anything, we are underestimating effect sizes compared with the general population. We did not have complete data on all individuals and chose to perform complete-case analysis, assuming data was missing at random. Reassuringly, results did not meaningfully change on using multiple imputation, but we cannot exclude the possibility that additional bias was introduced if missingness was associated with an imaging outcome. We used a binary measure of β-amyloid burden and therefore cannot exclude the possibility that small influences of vascular risk on β-amyloid deposition are not detected using this approach. Vascular risk intervention may modify associations, but it is challenging to account for in longitudinal observational work and was therefore not investigated. Participants were predominantly dementia-free at the end of data collection, and therefore we have not examined associations with cognition and dementia directly. However, brain volume,^43^ WMHV,^44^ and β-amyloid pathology^44^ are all associated with subsequent cognitive impairment, and it is reasonable to infer that the findings will have implications for future cognitive decline.

## Conclusions

Higher vascular risk is associated with smaller WBV and higher WMHV at age 69 to 71 years, with the strongest association seen with early adulthood vascular risk. There was no evidence that higher vascular risk influences cognitive health via β-amyloid deposition. The importance of elevated vascular risk in early adulthood should be recognized and appropriate lifestyle modification or other interventions considered.

## Notes

Supplement. **eMethods.** **eFigure 1.** Flowchart providing an overview of Insight 46 recruitment from the MRC NSHD and summary of imaging data available. **eFigure 2.** Scatter plots showing associations between FHS-CVS score at each age and white matter hyperintensity volume and whole brain volume at age 69-71 years. **eTable 1.** Comparison of characteristics between those included in WMHV analyses and those excluded due to missing covariate data and missing imaging data, in individuals without dementia **eTable 2.** Comparison of characteristics between those included in amyloid analyses and those excluded due to missing covariate data and missing imaging data, in individuals without dementia **eTable 3.** Comparison of characteristics between those included in brain volume analyses and those excluded due to missing covariate data and missing imaging data, in individuals without dementia **eTable 4.** Comparison of FHS-CVS between participants with available data for analysis in Insight 46 and those in the full cohort **eTable 5.** Associations between FHS cardiovascular risk scores at ages 36 (early adulthood), 53 (midlife) and 69 years (late-life) and whole brain volumes at age 69-71 years, adjusting for amyloid status and WMHV **eTable 6.** Associations between other covariates and brain outcome measures at age 69-71 years **eTable 7.** Associations between FHS cardiovascular risk scores at ages 36 (early adulthood), 53 (midlife) and 69 years (late-life) and WMHV, amyloid status, whole brain and mean hippocampal volumes at age 69-71 years using imputation for missing values
