# Supplementary material for: Associations Between Vascular Risk Across Adulthood and Brain Pathology in Late Life: Evidence From a British Birth Cohort
Source: JAMA Neurol. 2019 Nov 4;77(2):175–83. doi: 10.1001/jamaneurol.2019.3774 (PMC6830432; doi:10.1001/jamaneurol.2019.3774)
Supplement: Supplement. — eMethods. eFigure 1. Flowchart providing an overview of Insight 46 recruitment from the MRC NSHD and summary of imaging data available. eFigure 2. Scatter plots showing associations between FHS-CVS score at each age and white matter hyperintensity volume and whole brain volume at age 69-71 years. eTable 1. Comparison of characteristics between those included in WMHV analyses and those excluded due to missing covariate data and missing imaging data, in individuals without dementia eTable 2. Comparison of characteristics between those included in amyloid analyses and those excluded due to missing covariate data and missing imaging data, in individuals without dementia eTable 3. Comparison of characteristics between those included in brain volume analyses and those excluded due to missing covariate data and missing imaging data, in individuals without dementia eTable 4. Comparison of FHS-CVS between participants with available data for analysis in Insight 46 and those in the full cohort eTable 5. Associations between FHS cardiovascular risk scores at ages 36 (early adulthood), 53 (midlife) and 69 years (late-life) and whole brain volumes at age 69-71 years, adjusting for amyloid status and WMHV eTable 6. Associations between other covariates and brain outcome measures at age 69-71 years eTable 7. Associations between FHS cardiovascular risk scores at ages 36 (early adulthood), 53 (midlife) and 69 years (late-life) and WMHV, amyloid status, whole brain and mean hippocampal volumes at age 69-71 years using imputation for missing values [file jamaneurol-77-175-s001.pdf]

Supplementary Online Content

Lane CA, Barnes J, Nicholas JM, et al. Associations between vascular risk across adulthood and brain pathology in late life: evidence from a British birth cohort. *JAMA Neurol*. Published November 4, 2019. doi:10.1001/jamaneurol.2019.3774

eMethods.
eFigure 1. Flowchart providing an overview of Insight 46 recruitment from the MRC NSHD and summary of imaging data available.
eFigure 2. Scatter plots showing associations between FHS-CVS score at each age and white matter hyperintensity volume and whole brain volume at age 69-71 years.
eTable 1. Comparison of characteristics between those included in WMHV analyses and those excluded due to missing covariate data and missing imaging data, in individuals without dementia
eTable 2. Comparison of characteristics between those included in amyloid analyses and those excluded due to missing covariate data and missing imaging data, in individuals without dementia
eTable 3. Comparison of characteristics between those included in brain volume analyses and those excluded due to missing covariate data and missing imaging data, in individuals without dementia
eTable 4. Comparison of FHS-CVS between participants with available data for analysis in Insight 46 and those in the full cohort
eTable 5. Associations between FHS cardiovascular risk scores at ages 36 (early adulthood), 53 (midlife) and 69 years (late-life) and whole brain volumes at age 69-71 years, adjusting for amyloid status and WMHV
eTable 6. Associations between other covariates and brain outcome measures at age 69-71 years
eTable 7. Associations between FHS cardiovascular risk scores at ages 36 (early adulthood), 53 (midlife) and 69 years (late-life) and WMHV, amyloid status, whole brain and mean hippocampal volumes at age 69-71 years using imputation for missing values

This supplementary material has been provided by the authors to give readers additional information about their work.

© 2019 CA Lane et al. *JAMA Neurology.*

eMethods

Eligibility criteria for Insight 46 were based on maximising the life course data available for analysis, and were intended to avoid *a priori* decisions as to who might be at risk of cognitive decline. MRC NSHD study members were recruited from those who attended a clinic-based assessment age 60-64 years, had previously indicated willingness to attend a clinic visit in London and for whom relevant data in childhood and adulthood were available, including at least one BP, height and weight measurement, and recorded smoking status from ages 36, 43, 53 and 60-64 years. Exclusion criteria were limited to contraindications to MRI or PET including, but not limited to, claustrophobia, metallic implants such as pacemakers, or research scans within the last year that would result in an individual exceeding acceptable mandated yearly radiation exposures.[1]

In a sensitivity analysis, multiple imputation was used for the 66 individuals excluded because of missing vascular covariate data. Under the assumption that vascular risk factors (systolic blood pressure, body mass index, anti-hypertensive usage, smoking and diabetic status) from which the FHS-CVS were derived were missing at random, missing vascular variables at the three time-points were imputed by chained equations using the *Mi* package in Stata 14.1. Imputations were carried out separately for each outcome, with each imputation model including all predictor variables and the outcome variable (plus available vascular risk factor variables at the other ages). 50 imputed datasets were generated for each outcome. Linear regression was used as the imputation model for systolic blood pressure and body mass index and logistic regression was used as the imputation model for anti-hypertensive usage, smoking and diabetic status. These imputed vascular risk factor values were then used to derive 50 imputations for FHS-CVS scores at each age. One FHS-CVS score could not be imputed at age 36 because the individual's diabetes status could not be imputed. The models for each outcome were then run for each set of imputed FHS-CVS scores and results combined using Rubin's rules.

eResults

eFigure 1 Flowchart providing an overview of Insight 46 recruitment from the MRC NSHD and summary of imaging data available.

Figure adapted with permission from James *et al*[1] under the terms of the Creative Commons Attribution 4.0 International License(http://creativecommons.org/licenses/by/4.0/). BaMoS, Bayesian Model Selection; MRI, magnetic resonance imaging; MCI, mild cognitive impairment; NSHD, National Survey of Health and Development; PET, positron emission tomography; QC, quality control; WMHV, white matter hyperintensity volume.

© 2019 CA Lane et al. *JAMA Neurology.*

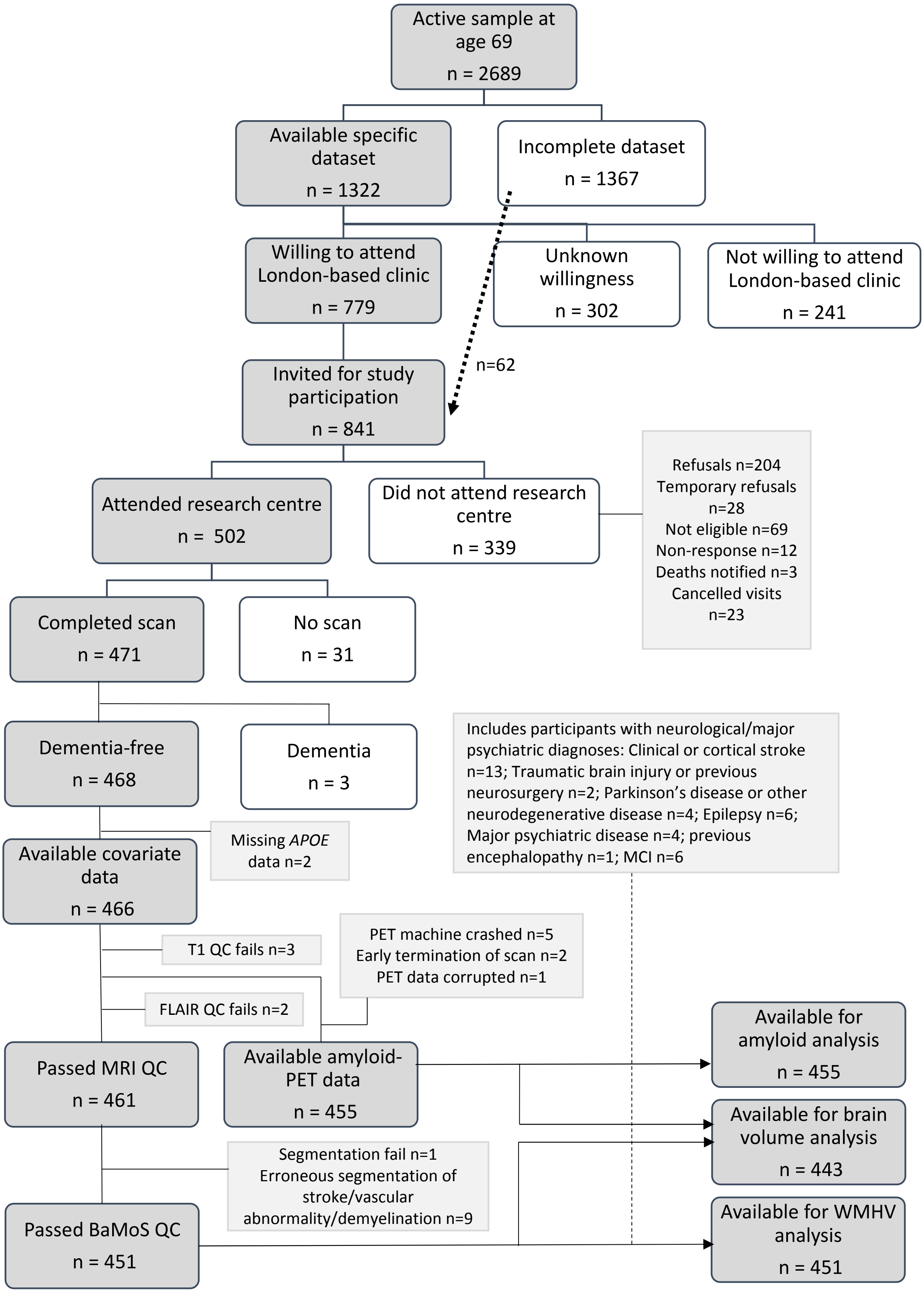

© 2019 CA Lane et al. *JAMA Neurology.*

eTable 1 Comparison of characteristics between those included in WMHV analyses and those excluded due to missing covariate data and missing imaging data, in individuals without dementia

*At age 36: 38 individuals were missing BMI, 40 missing SBP, 36 missing smoking status, 37 missing diabetic status and anti-hypertensive medication usage.
[†] At age 53: 9 individuals were missing BMI, 12 missing SBP, 8 missing smoking status and anti-hypertensive usage and 1 missing diabetic status.
[‡] At age 69: 7 individuals were missing BMI, 6 missing SBP, 4 missing diabetic status and 12 missing anti-hypertensive usage.
[**] Missing imaging data was due to failure to tolerate scan (31), scan QC failure (5) or BaMoS QC failure (10)
BMI, body mass index; FHS-CVS, Framingham Heart Study – Cardiovascular risk score; IQR, interquartile range; NA, not applicable; QC, quality control; SBP systolic blood pressure; SD, standard deviation; SEP, socioeconomic position; TIV, total intracranial volume; WMHV, white matter hyperintensity volume

		**Age 36 years**			**Age 53 years**			**Age 69 years**		
**Characteristic**		**Included, n=407**	**Excluded, missing covariate data[*],n=44**	**Excluded, missing imaging data[**],n=46**	**Included, n=438**	**Excluded, missing covariate data[†], n=13**	**Excluded, missing imaging data[**],n=46**	**Included, n=438**	**Excluded, missing covariate data[‡], n=13**	**Excluded, missing imaging data[**], n=46**
**Age in years, mean (SD)**		36.3 (0.2)	36.3 (0.2)	36.3 (0.1)	53.4 (0.2)	53.4 (0.1)	53.5 (0.2)	69.5 (0.2)	69.5 (0.3)	69.5 (0.2)
**Male, n (%)**		208 (51.1)	23 (52.3)	22 (47.8)	225 (51.4)	6 (46.2)	22 (47.8)	227 (51.8)	4 (30.8)	22 (47.8)
**Adult SEP, n (%)**	**Non-manual**	349 (85.8)	32 (72.7)	41 (89.1)	372 (84.9)	9 (69.2)	41 (89.1)	374 (85.4)	7 (53.9)	41 (89.1)
	**Manual**	58 (14.3)	12 (27.3)	5 (10.9)	66 (15.1)	4 (30.8)	5 (10.9)	64 (14.6)	6 (46.2)	5 (10.9)
***APOE*-ε4 status (1 or 2), n (%)**		125 (30.7)	9 (20.5)	12 (26.1)	127 (29.0)	7 (53.9)	12 (26.1)	127 (29.0)	7 (53.9)	12 (26.1)
**SBP, mean (SD), mmHg**		120.1 (13.8)	117.7 (17.1)	120.8 (13.0)	133.4 (19.1)	168 (NA)	133.0 (17.9)	132.4 (16.1)	124.9 (17.9)	132.9 (15.5)
**Anti-hypertensive medication, n (%)**		7 (1.7)	0 (0.0)	0 (0.0)	48 (11.0)	1 (20.0)	8 (17.8)	171 (39.0)	1 (100.0)	21 (46.7)
**BMI, mean (SD)**		23.7 (3.1)	23.7 (3.5)	24.3 (3.2)	26.9 (4.0)	33.0 (1.4)	28.1 (4.4)	27.5 (4.4)	27.0 (5.4)	30.1 (5.1)
**Current smoker, n (%)**		81 (19.9)	1 (12.5)	9 (20.9)	41 (9.4)	1 (20.0)	6 (13.3)	16 (3.7)	0 (0.0)	2 (4.4)
**Diabetes, n (%)**		1 (0.25)	0 (0)	0 (0.0)	13 (3.0)	0 (0.0)	1 (2.2)	46 (10.5)	2 (22.2)	6 (13.3)
**FHS-CVS (%), median (IQR)**		2.7 (1.5, 3.6)	NA	2.8 (1.5, 3.5)	10.8 (6.5, 15.6)	NA	11.7 (7.1, 14.9)	24.2 (14.9, 34.9)	NA	24.1 (16.8, 38.2)
**Scanning age in years, mean (SD)**		70.7 (0.7)	70.8 (0.6)	NA	70.7 (0.7)	71.0 (0.8)	NA	70.7 (0.7)	71.0 (0.7)	NA
**Global WMHV (ml), median (IQR)**		3.2 (1.6, 7.0)	2.2 (1.0, 3.6)	NA	3.1 (1.6, 6.6)	6.0 (1.8, 8.0)	NA	3.0 (1.6, 6.8)	4.1 (2.1, 6.2)	NA
**TIV (ml), mean (SD)**		1434.5 (132.0)	1427.7 (137.4)	NA	1433.6 (132.7)	1440.5 (129.8)	NA	1435.7 (131.7)	1368.7 (147.3)	NA

© 2019 CA Lane et al. *JAMA Neurology.*

eTable 2 Comparison of characteristics between those included in amyloid analyses and those excluded due to missing covariate data and missing imaging data, in individuals without dementia

*At age 36: 39 individuals were missing BMI, 41 missing SBP, 37 missing smoking status and 38 missing diabetic status and anti-hypertensive medication usage.

† At age 53: 9 missing BMI, 13 missing SBP, 8 missing smoking status and anti-hypertensive medication and 1 missing diabetic status.

‡ At age 69: 7 missing BMI, 6 missing SBP, 4 missing diabetic status and 12 missing anti-hypertensive medication.

** Missing imaging data was due to failure to tolerate scan (31), PET processing failure (8) or scan T1 QC failure (3).

BMI, body mass index; FHS-CVS, Framingham Heart Study – Cardiovascular risk score; IQR, interquartile range; n, number; NA, not applicable; QC, quality control; SBP systolic blood pressure; SD, standard deviation; SEP, socioeconomic position

		Age 36 years			Age 53 years			Age 69 years		
**Characteristic**		**Included, n=410**	**Excluded, missing covariate data*,n=45**	**Excluded, missing imaging data**,n=42**	**Included, n=441**	**Excluded, missing covariate data†, n=14**	**Excluded, missing imaging data**,n=42**	**Included, n=442**	**Excluded, missing covariate data‡, n=13**	**Excluded, missing imaging data**, n=42**
**Age in years, mean (SD)**		36.3 (0.2)	36.3 (0.2)	36.3 (0.1)	53.4 (0.2)	53.4 (0.2)	53.5 (0.2)	69.5 (0.2)	69.5 (0.3)	69.4 (0.2)
**Male, n (%)**		207 (50.5)	23 (51.1)	23 (54.8)	223 (50.6)	7 (14.0)	23 (54.8)	226 (51.1)	4 (30.8)	23 (54.8)
**Adult SEP, n (%)**	**Non-manual**	353 (86.1)	33 (73.3)	36 (85.7)	376 (85.3)	10 (71.4)	36 (85.7)	379 (85.8)	7 (53.9)	36 (85.7)
	**Manual**	57 (13.9)	12 (26.7)	6 (14.3)	65 (14.7)	4 (28.6)	6 (14.3)	63 (14.3)	6 (46.2)	6 (14.3)
***APOE*-ε4 status (1 or 2), n (%)**		122 (29.8)	9 (20.0)	15 (35.7)	123 (27.9)	8 (57.1)	15 (35.7)	124 (28.1)	7 (53.9)	15 (35.7)
**SBP, mean (SD), mmHg**		120.2 (13.8)	117.7 (17.1)	120.0 (12.6)	133.5 (19.3)	168 (NA)	132.0 (15.8)	132.4 (16.0)	124.9 (17.9)	132.8 (16.3)
**Anti-hypertensive medication, n (%)**		7 (1.7)	0 (0.0)	0 (0.0)	51 (11.6)	1 (16.7)	5 (12.2)	172 (38.9)	1 (100.0)	20 (48.8)
**BMI, mean (SD)**		23.7 (3.1)	23.7 (3.5)	24.5 (3.5)	26.9 (4.1)	32.1 (2.3)	27.7 (4.3)	27.6 (4.4)	27.0 (5.4)	29.7 (5.1)
**Current smoker, n (%)**		80 (19.5)	1 (12.5)	10 (25.0)	41 (9.3)	1 (16.7)	6 (14.6)	16 (3.6)	0 (0.0)	2 (4.8)
**Diabetes, n (%)**		1 (0.2)	0 (0.0)	0 (0.0)	13 (3.0)	0 (0.0)	1 (2.4)	47 (10.6)	2 (22.2)	5 (12.2)
**FHS-CVS (%), median (IQR)**		2.6 (1.5, 3.6)	NA	2.9 (1.5, 3.7)	10.7 (6.5, 15.6)	NA	12.6 (6.8, 15.4)	24.2 (14.9, 34.9)	NA	25.5 (19.1, 38.5)
**Scanning age in years, mean (SD)**		70.7 (0.7)	70.8 (0.6)	NA	70.7 (0.7)	71.1 (0.8)	NA	70.7 (0.7)	71.0 (0.7)	NA
**Amyloid positive, n (%)**		74 (18.1)	9 (20.0)	NA	82 (18.6)	1 (7.1)	NA	81 (18.3)	2 (15.4)	NA

© 2019 CA Lane et al. *JAMA Neurology.*

eTable 3 Comparison of characteristics between those included in brain volume analyses and those excluded due to missing covariate data and missing imaging data, in individuals without dementia

* At age 36: 38 individuals were missing BMI, 40 missing SBP, 36 missing smoking status and 37 missing diabetic status and anti-hypertensive medication usage.

† At age 53: 9 individuals were missing BMI, 12 missing SBP, 8 missing smoking status and anti-hypertensive medication and 1 missing diabetic status.

‡ At age 69: 7 individuals were missing BMI, 6 missing SBP, 4 missing diabetic status and 12 missing anti-hypertensive medication.

** Missing imaging data was due to failure to tolerate scan (31), scan QC failure (5), or unavailable amyloid (8) or WMHV status (10).

BMI, body mass index; FHS-CVS, Framingham Heart Study – Cardiovascular risk score; HV, hippocampal volume; IQR, interquartile range; n, number; NA, not applicable; QC, quality control; SBP systolic blood pressure; SD, standard deviation; SEP, socioeconomic position; TIV, total intracranial volume; WBV, whole brain volume

		Age 36 years			Age 53 years			Age 69 years		
**Characteristic**		**Included, n=399**	**Excluded, missing covariate data*,n=44**	**Excluded, missing imaging data**,n=54**	**Included, n=430**	**Excluded, missing covariate data†, n=13**	**Excluded, missing imaging data**,n=54**	**Included, n=430**	**Excluded, missing covariate data‡, n=13**	**Excluded, missing imaging data**, n=54**
**Age in years, mean (SD)**		36.3 (0.2)	36.3 (0.2)	36.3 (0.1)	53.4 (0.2)	53.4 (0.1)	53.5 (0.2)	69.5 (0.2)	69.5 (0.3)	69.4 (0.2)
**Male, n (%)**		202 (50.6)	23 (52.3)	28 (51.9)	219 (50.9)	6 (46.2)	28 (51.9)	221 (51.4)	4 (30.8)	28 (51.9)
**Adult SEP, n (%)**	**Non-manual**	342 (85.7)	32 (72.7)	48 (88.9)	9 (69.2)	9 (69.2)	48 (88.9)	7 (53.9)	7 (53.9)	48 (88.9)
	**Manual**	57 (14.3)	12 (27.3)	6 (11.1)	4 (30.8)	4 (30.8)	6 (11.1)	6 (46.2)	6 (46.2)	6 (11.1)
***APOE*-ε4 status (1 or 2), n (%)**		119 (29.8)	9 (20.5)	18 (33.3)	121 (28.1)	7 (53.9)	18 (33.3)	121 (28.1)	7 (53.9)	18 (33.3)
**SBP, mean (SD), mmHg**		120.1 (13.8)	117.7 (17.1)	121.1 (12.7)	133.3 (19.2)	168 (NA)	134.3 (17.5)	132.4 (16.2)	124.9 (17.9)	132.8 (15.1)
**Anti-hypertensive medication, n (%)**		7 (1.8)	0 (0.0)	0 (0.0)	46 (10.7)	1 (20.0)	10 (18.9)	163 (37.9)	1 (100.0)	29 (54.7)
**BMI, mean (SD)**		23.7 (3.1)	23.7 (3.5)	24.3 (3.3)	26.9 (4.1)	33.0 (1.4)	27.8 (4.2)	27.5 (4.4)	27.0 (5.4)	29.6 (5.0)
**Current smoker, n (%)**		79 (19.8)	1 (12.5)	11 (21.6)	41 (9.5)	1 (20.0)	6 (11.3)	16 (3.7)	0 (0.0)	2 (3.7)
**Diabetes, n (%)**		1 (0.25)	0 (0.0)	0 (0.0)	13 (3.0)	0 (0.0)	1 (1.9)	46 (10.7)	2 (22.2)	6 (11.3)
**FHS-CVS (%), median (IQR)**		2.6 (1.5, 3.6)	NA	2.8 (1.5, 3.6)	10.7 (6.4, 15.6)	NA	12.1 (7.8, 15.7)	24.0 (14.6, 34.9)	NA	26.0 (19.1, 38.2)
**Scanning age in years, mean (SD)**		70.7 (0.7)	70.8 (0.6)	NA	70.7 (0.7)	71.0 (0.8)	NA	70.7 (0.7)	71.0 (0.7)	NA
**WBV (mls), mean (SD)**		1101.4 (98.4)	1087.5 (97.3)	NA	1099.4 (98.5)	1119.9 (91.1)	NA	1101.6 (97.5)	1049.0 (113.1)	NA

© 2019 CA Lane et al. *JAMA Neurology.*

**Mean HV (mls), mean (SD)**	3.1 (0.3)	3.1 (0.3)	NA	3.1 (0.3)	3.1 (0.2)	NA	3.1 (0.3)	3.0 (0.4)	NA
**TIV (mls), mean (SD)**	1432.2 (131.4)	1427.7 (137.4)	NA	1431.5 (132.1)	1440.4 (129.8)	NA	1433.7 (131.1)	1368.7 (147.3)	NA

eTable 4 Comparison of FHS-CVS between participants with available data for analysis in Insight 46 and those in the full cohort Median and interquartile ranges are stated, together with number of participants with available data at each time point. NSHD, National Survey for Health and Development; FHS-CVS, Framingham Heart Study - Cardiovascular risk score

Age	Insight 46	Full NSHD cohort
**36 years**	2.7 (1.5, 3.6)<br>n=418	2.9 (1.7, 4.3)<br>n=2801
**53 years**	10.9 (6.7, 15.6)<br>n=449	12.2 (7.6, 18.8)<br>n=2413
**69 years**	24.3 (14.9, 34.9)<br>n=450	25.0 (15.3, 36.5)<br>n=1601

© 2019 CA Lane et al. *JAMA Neurology.*

eFigure 2 Scatter plots showing associations between FHS-CVS score at each age and white matter hyperintensity volume and whole brain volume at age 69-71 years.

Scatter plots show associations between FHS-CVS scores at ages 36, 53 and 69 years, and global WMHV (top panels) and adjusted WBV (bottom panels) at age 69-71 years. Whole brain volume is adjusted for sex, age at scanning and total intracranial volume. Lines of best fit are plotted with 95% confidence intervals (CI). FHS-CVS, Framingham Cardiovascular risk score; WBV, whole brain volume; WMHV, white matter hyperintensity volume.

© 2019 CA Lane et al. *JAMA Neurology.*

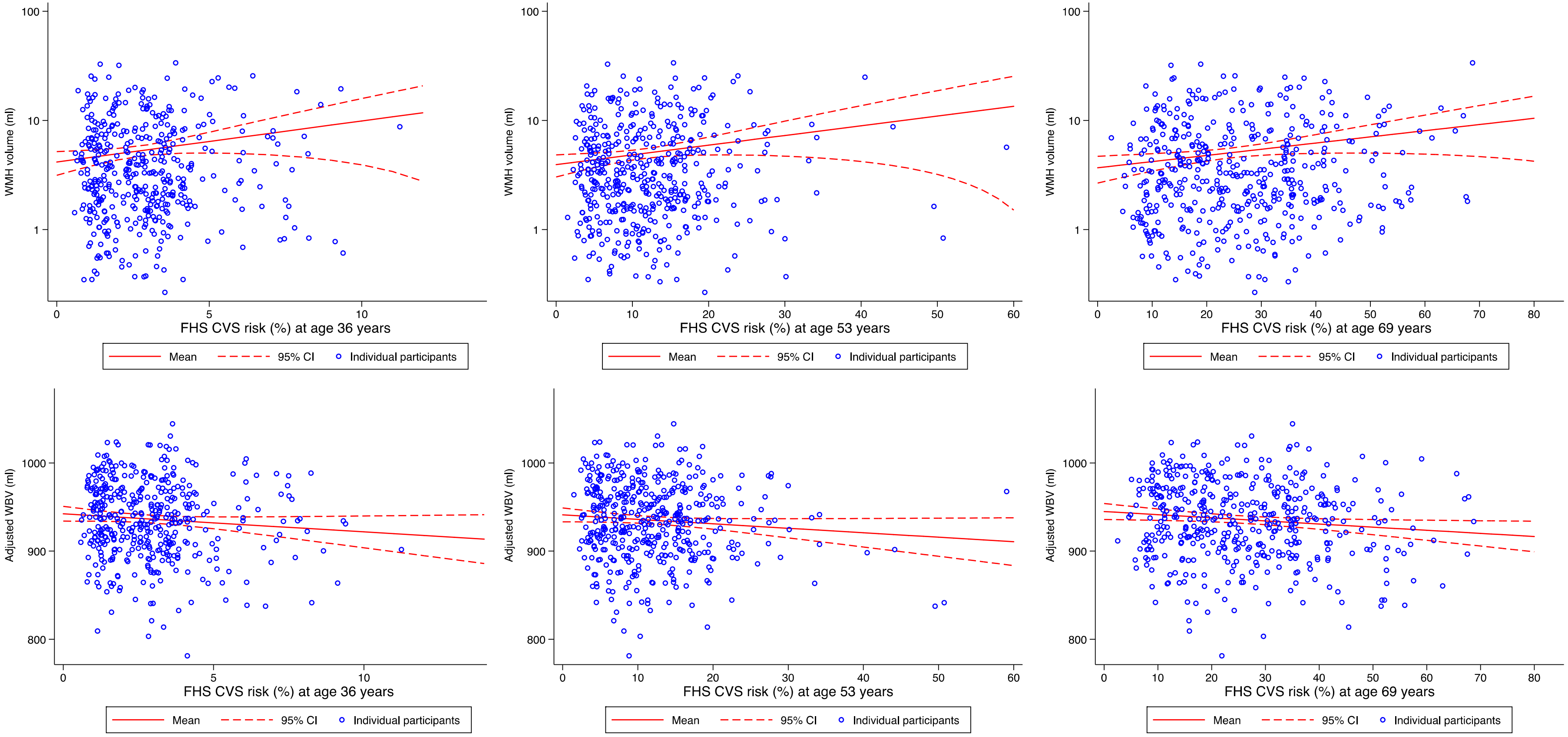

© 2019 CA Lane et al. *JAMA Neurology.*

eTable 5 Associations between FHS cardiovascular risk scores at ages 36 (early adulthood), 53 (midlife) and 69 years (late-life) and whole brain volumes at age 69-71 years, adjusting for amyloid status and WMHV. Coefficients represent the change in WBV per 1% increase in FHS-CVS. All models are adjusted for sex, age at time of scanning, APOE-ε4 carrier status, adult SEP, TIV, amyloid status and global WMHV. CI, confidence interval; FHS-CVS, Framingham Heart Study cardiovascular risk score; SEP, socioeconomic position; TIV, total intracranial volume; WBV, whole brain volume; WMHV, white matter hyperintensity volume.

		**WBV (ml)**<br>Age 36, n=399<br>Age 53, n=430<br>Age 69, n=430	
		β coefficient (95% CI)	*P value*
**FHS cardiovascular score**	**Age 36**	**-3.7 (-7.1, -0.3)**	**0.034**
	**Age 53**	**-0.8 (-1.5, -0.07)**	**0.031**
	**Age 69**	**-0.6 (-1.1, -0.2)**	**0.003**

© 2019 CA Lane et al. *JAMA Neurology.*

eTable 6 Associations between other covariates and brain outcome measures at age 69-71 years. All associations stated use the FHS-CVS models at age 36 as representative examples. BMI, body mass index; CI, confidence interval; FHS-CVS, Framingham Heart Study cardiovascular risk score; HV, hippocampal volume; OR, odds ratio; TIV, total intracranial volume; WBV, whole brain volume; WMHV, white matter hyperintensity volume.

	**WMHV Exponentiated coefficient (95%CI)**	***P* value**	**Amyloid Adjusted OR (95% CI)**	***P* value**	**WBV β coefficient (95% CI)**	***P* value**	**Mean HV β coefficient (95% CI)**	***P* value**
**Sex (male)**	**0.62 (0.44, 0.86)**	**0.005**	1.14 (0.55, 2.36)	0.72	**-14.7 (-29.0, -0.5)**	**0.043**	**0.11 (0.02, 0.20)**	**0.017**
***APOE*-ε4 carrier**	1.08 (0.86, 1.36)	0.49	**5.16 (3.02, 8.81)**	**<0.001**	8.0 (-1.5, 17.5)	0.10	-0.01 (-0.07, 0.05)	0.65
**TIV**	1.00 (1.00, 1.00)	0.19			**0.7 (0.7, 0.8)**	**<0.001**	**0.001 (0.001, 0.001)**	**<0.001**
**Scanning age**	**1.18 (1.01, 1.37)**	**0.039**	0.98 (0.66, 1.45)	0.91	**-11.5 (-17.9, -5.0)**	**0.001**	**-0.04 (-0.08, -0.002)**	**0.041**
**Adult SEP (manual)**	1.01 (0.75, 1.37)	0.94	0.71 (0.31, 1.63)	0.42	11.5 (-0.9, 24.0)	0.070	-0.009 (-0.09, 0.07)	0.82

eTable 7 Associations between FHS cardiovascular risk scores at ages 36 (early adulthood), 53 (midlife) and 69 years (late-life) and WMHV, amyloid status, whole brain and mean hippocampal volumes at age 69-71 years using imputation for missing values. Coefficients represent the change in imaging outcomes measures per 1% increase in FHS-CVS. All models are adjusted for sex, age at time of scanning, *APOE*-ε4 carrier status, adult SEP, and (where appropriate) TIV. CI, confidence interval; HV, hippocampal volume; n, number; SEP, socioeconomic position; TIV, total intracranial volume; WBV, whole brain volume; WMHV, white matter hyperintensity volume.

		**WMHV (ml)**<br>Age 36, n=450<br>Age 53, n=451<br>Age 69, n=451		**Amyloid status**<br>Age 36, n=454<br>Age 53, n=455<br>Age 69, n=455		**WBV (ml)**<br>Age 36, n=442<br>Age 53, n=443<br>Age 69, n=443		**Mean HV (ml)**<br>Age 36, n=442<br>Age 53, n=443<br>Age 69, n=443	
		Exponentiated coefficient (95% CI)	***P value***	***Adjusted odds ratio (95% CI)***	***P value***	***β coefficient (95% CI)***	***P value***	***β coefficient (95% CI)***	***P value***
**FHS cardiovascular score**	**Age 36**	**1.09 (1.005, 1.18)**	**0.037**	1.00 (0.81, 1.23)	0.98	-3.3 (-6.5, 0.03)	0.052	**-0.03 (-0.05, -0.004)**	**0.020**
	**Age 53**	**1.02 (1.003, 1.04)**	**0.022**	0.97 (0.92, 1.02)	0.20	**-0.7 (-1.4, -0.02)**	**0.043**	-0.0002 (-0.005, 0.004)	0.95
	**Age 69**	**1.01 (1.003, 1.02)**	**0.011**	0.99 (0.96, 1.01)	0.40	**-0.6 (-1.0, -0.2)**	**0.004**	0.0007 (-0.0021, 0.0034)	0.64

© 2019 CA Lane et al. *JAMA Neurology.*

References

1. James SN, Lane CA, Parker TD, et al. Using a birth cohort to study brain health and preclinical dementia: Recruitment and participation rates in Insight 46. BMC Res Notes. 2018;11:885.

© 2019 CA Lane et al. *JAMA Neurology.*
